# Construction and assessment of a predictive framework for Enterobacterales bloodstream infection in real-world adult onco-hematology patients using Lasso-Cox regression

**DOI:** 10.1128/aac.00973-25

**Published:** 2025-10-17

**Authors:** Yuqing Cui, Xiaomeng Feng, Jieru Wang, Li Liu, Lining Zhang, Qingsong Lin, Ling Pan, Sisi Zhen, Yuping Fan, Xin Chen, Tingting Zhang, Yingchang Mi, Zhijian Xiao, Erlie Jiang, Mingzhe Han, Jianxiang Wang, Sizhou Feng

**Affiliations:** 1 State Key Laboratory of Experimental Hematology, National Clinical Research Center for Blood Diseases, Haihe Laboratory of Cell Ecosystem, Institute of Hematology & Blood Diseases Hospital, Chinese Academy of Medical Sciences & Peking Union Medical College, Tianjin, China; 2 Tianjin Institutes of Health Science, Tianjin, China; University of Fribourg, Fribourg, Switzerland

**Keywords:** *Enterobacterales* bloodstream infection, Lasso-Cox regression, onco-hematology, prognostic model, 30-day mortality

## Abstract

Bloodstream infections (BSIs) caused by *Enterobacterales*, particularly *Escherichia coli* (EC) and *Klebsiella pneumoniae* (KP), are the most prevalent infectious issues in individuals with hematologic malignancies (HM). Prognostic tools integrating pathogen-specific risks are urgently needed to optimize early clinical decision-making. This retrospective cohort study analyzed 1,299 HM patients with EC- or KP-BSI (2017–2023). Patients were randomly allocated to training (*n* = 909) and validation (*n* = 390) cohorts. Prognostic variables were selected through Lasso regression, followed by Cox proportional hazards modeling to construct a 30-day mortality prediction nomogram. Model performance was rigorously validated using receiver operating characteristic curves (AUCs), calibration curves, and decision-curve analysis. The cohort comprised 653 EC-BSI and 646 KP-BSI cases, predominantly with acute myeloid leukemia (54.3%). Pulmonary/skin-soft tissue infection origins, hematopoietic stem cell transplantation, concurrent septic shock/pneumonia, hypoproteinemia, prolonged neutropenia before BSI, and carbapenem-resistant strains emerged as independent predictors of mortality. Conversely, tumor consolidation stage status and ceftazidime-avibactam-containing regimens were predictive of survival benefit. The model demonstrated high predictive accuracy (AUC 0.881), excellent calibration, and superior clinical utility. We developed a simple model using routine clinical parameters that predicts 30-day mortality in HM patients with EC- or KP-BSI, guiding therapy without complex biomarkers.

## INTRODUCTION

Recent advancements in the treatment of hematological malignancies (HMs) have significantly improved remission and survival rates. However, due to the severe immunosuppression and prolonged neutropenia during treatment, HM patients are more susceptible to immune dysfunction, which increases the susceptibility of patients to infections, particularly bloodstream infections (BSIs). The incidence of BSI in HM patients varies between 11% and 38% (1), fatality rates spanning from 10% to 34% (2).

Among the most common pathogens in these infections are *Enterobacterales*, particularly *Escherichia coli* (EC) and *Klebsiella pneumoniae* (KP), which are frequently identified in blood cultures (3). Previous studies have highlighted several factors associated with poor BSI outcomes in HM patients, including older age, uncontrolled or relapsed malignancy, prolonged neutropenia, inadequate antibiotic therapy, low serum albumin levels, the presence of multidrug-resistant (MDR) organisms, and central venous catheter (CVC) placement (4–6). Accurate prognostic stratification of BSIs in patients with HM remains a critical unmet need. While EC and KP dominate the microbiological landscape of HM-associated BSIs, existing prediction models seldom account for pathogen-specific risk modifiers. Regional disparities in antimicrobial resistance patterns further challenge the generalizability of existing models, which are often derived from single-center cohorts with insufficient sample sizes. These gaps underscore the necessity for a pathogen-adaptive prognostic framework that incorporates both host vulnerability and evolving resistance profiles.

Real-world studies, especially those with large sample sizes, are increasingly recognized for their ability to provide valuable clinical evidence. This retrospective study focuses on a large cohort of HM patients with BSI caused by EC or KP. By integrating host factors, treatment parameters, and resistance profiles, we aim to build a model that can predict the risk of 30-day mortality. Our intention is to furnish clinicians with a tool for the early recognition of high-risk patients, so as to enable timely actions that can decrease the mortality associated with BSI.

## MATERIALS AND METHODS

### Enrolled participants

This retrospective analysis encompassed 1,299 patients aged 14 years or older, who were hospitalized at the Institute of Hematology & Blood Diseases Hospital (Chinese Academy of Medical Sciences) from January 2017 to December 2023. All patients had positive blood cultures for EC or KP. The exclusion criteria were (i) infection with multiple pathogens, or (2) age <14 years. To enhance the reliability of the predictive model, participants were randomly divided into training (70%) and validation (30%) sets. The hospital’s ethics committee gave approval for the study.

### Data collection

We collected baseline characteristics, laboratory results, and treatment information, including patient demographics (age and gender), primary disease, tumor stage (consolidation stage or not), hematopoietic stem cell transplantation (HSCT) status, infection sources, concurrent infections, neutrophil count, serum albumin levels, and details of antimicrobial therapy administered before infection onset. Additionally, we recorded the type of BSI pathogens and antimicrobial treatment regimens used during the BSI episode. The main endpoint was all-cause mortality within 30 days.

### Definitions

Remission status was assessed according to recent clinical guidelines for acute leukemia (7, 8), lymphoma (9), and myelodysplastic syndromes (10). BSI due to EC or KP was determined when either of these pathogens was detected in the blood cultures of patients with suspected infection. The origin of bacteremia was determined comprehensively by identifying concurrent active infection sites or isolating pathogens from specimens clinically obtained within 24 h before bacteremia onset. Septic shock was defined as the need for vasopressor support to maintain a mean arterial pressure of no more than 65 mmHg or a serum lactate level ≥2 mmol/L (11). Concurrent pneumonia was strictly defined as secondary pulmonary infection that developed during the infection course, where the BSI originated from non-pulmonary sites. Appropriate empirical therapy was considered as the use of antibiotics that demonstrated *in vitro* activity against the identified pathogen, within 24 h of infection recognition or clinical suspicion, and administered at an appropriate dosage and route of administration according to the Infectious Diseases Society of America 2024 guidance recommendations (12). Profound neutropenia was described as an absolute neutrophil count (ANC) less than 0.1 × 10⁹/L. Meanwhile, prolonged neutropenia was characterized as neutropenia that endured for 14 days. Antibiotic exposure was characterized as antibiotics use for over 72 h within the 90-day period preceding the BSI diagnosis.

### Microbiological testing

Pathogen detection was conducted utilizing the VITEK 2 Compact blood culture system. Antimicrobial susceptibility was gaged with the disc diffusion method. Detection of carbapenem-resistant organisms was carried out by the modified carbapenem inactivation method, both following the Clinical and Laboratory Standards Institute (CLSI) M100 guidelines.

### Statistical analysis

Data analysis was carried out using R software (version 4.3.3). For continuous variables, they were characterized by medians along with interquartile ranges (IQRs), and compared using independent *t*-tests or Mann-Whitney U tests. Categorical variables were shown as frequencies and percentages, and χ or Fisher’s exact tests were used for making comparisons. Lasso regression in R was first applied for the initial selection of variables. Subsequently, the chosen variables were subjected to further analysis through Cox regression. Only those variables reaching statistical significance (*P* < 0.1) were incorporated into the development of the final predictive model. We used inverse probability of treatment weighting (IPTW), adjusting for key covariates, to balance baseline characteristics between treatment groups. To evaluate the accuracy and consistency of the nomogram model, calibration plots and the concordance index (C-index) were employed. A C-index <0.5 indicates no predictive value, 0.5 < C-index ≤ 0.7 suggests poor accuracy, and a C-index >0.7 indicates good predictive performance. Finally, decision curve analysis (DCA) was used to assess the clinical utility of the model.

## RESULTS

### Patient characteristics

**Fig 1:**
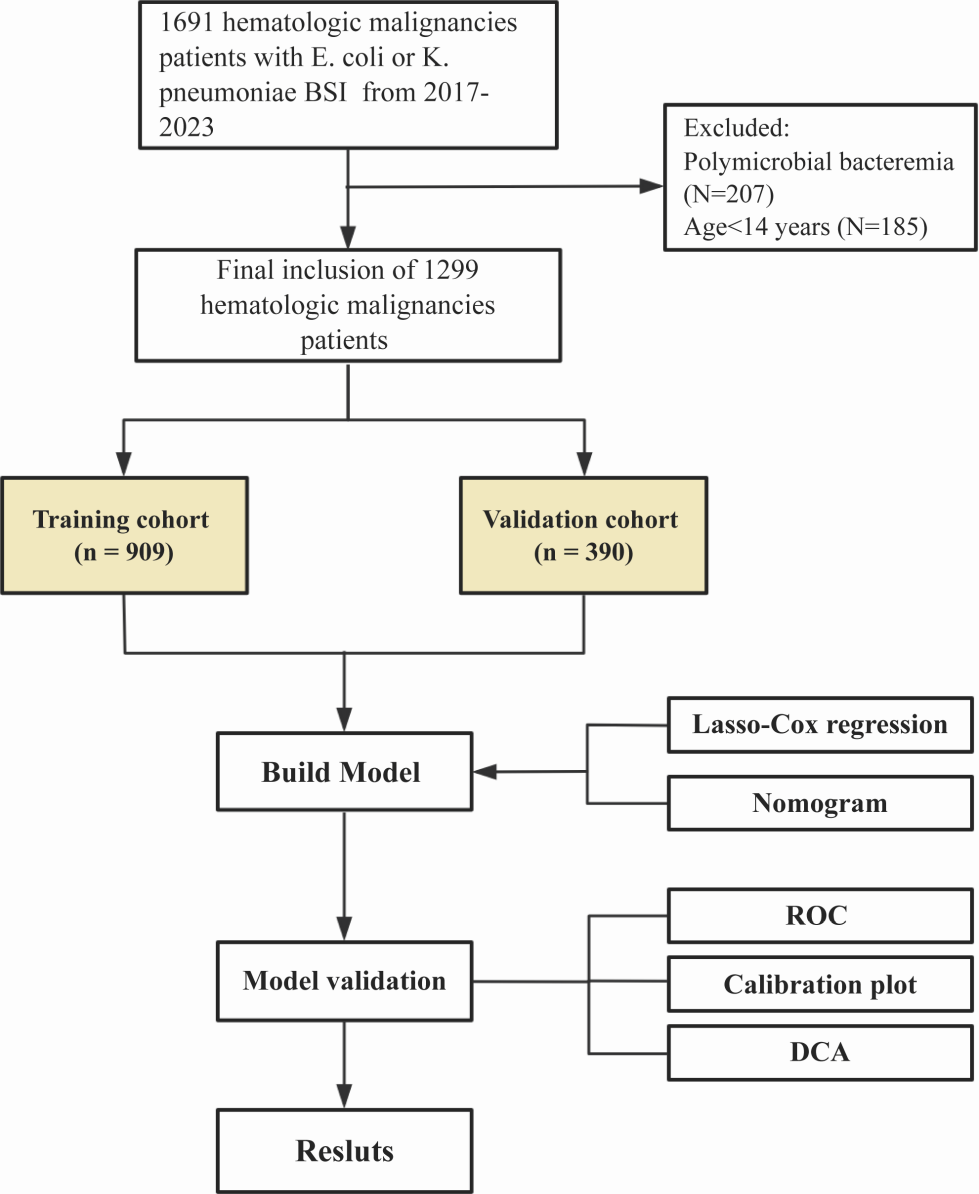
Flowchart of the study design. The study cohort was randomly split into training and validation sets to develop and assess a predictive nomogram using Lasso-Cox regression.

This study included 1,299 HM patients with BSIs caused by EC or KP (Fig. 1). Of these, 653 patients (50.27%) had EC bacteremia, while 646 patients (49.73%) had KP bacteremia. The cohort was made up of 706 patients suffering from acute myeloid leukemia (AML) and 379 patients with acute lymphoblastic leukemia (ALL). The median age of these patients was 43 years, with 53.73% being male. Among the patients, 685 were in the tumor remission phase, and 15.78% had undergone hematologic stem cell transplantation (HSCT). The majority of patients (55.89%) had no identified source of infection. Profound neutropenia was present in 71.82% of the patients. Additionally, 104 patients had carbapenem-resistant infections, and 84 received inappropriate empirical antibiotic therapy within 48 h (IET48H) after infection onset. The median duration of neutropenia prior to infection was 3.00 days (IQR 1.00–6.00), and following BSI, it was 5.00 days (IQR 3.00–9.00). Among them, 97 had prolonged neutropenia prior to the BSI, while 13.47% (175/1299) experienced prolonged neutropenia after BSI. Following the completion of antibiotic therapy, 196 patients remained neutropenic. The 30-day mortality rate for EC-BSI and KP-BSI cases was 5.62%. Significantly, the mortality was greater among patients with carbapenem-resistant isolates than those with non-resistant ones (19.23% vs. 4.44%, *P* < 0.001), underscoring the critical impact of antimicrobial resistance on patient outcomes (Fig. S1.

### Prediction model development using Lasso-Cox regression

**Fig 2:**
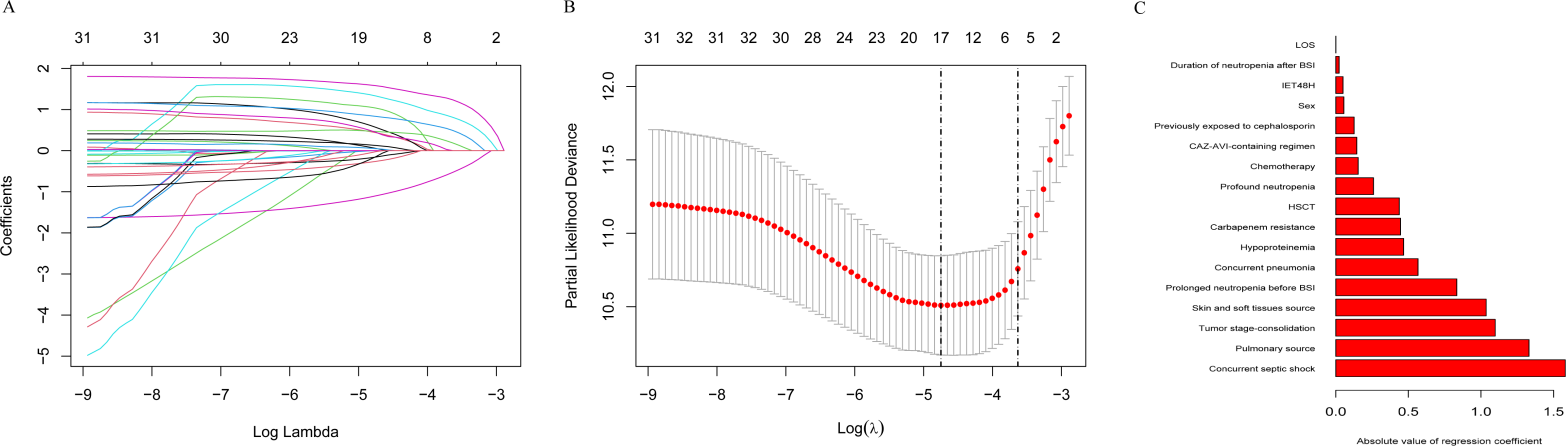
Feature selection and coefficient estimation using least absolute shrinkage and selection operator (Lasso) regression with 10-fold cross-validation. (**A**) Feature selection process. The Y-axis represents the magnitude of the regression coefficients for each predictor variable. The X-axis represents the logarithm of the regularization parameter lambda (λ) in the Lasso model. As the penalty increases, the variables retained in the model (those converging to the right) are considered the most important predictors. (**B**) Selection of the optimal tuning parameter. The partial likelihood deviance is plotted against the logarithm of the regularization parameter (λ). Vertical dashed lines indicate the optimal λ values chosen by the minimum deviance criterion (left) and the 1-standard-error rule (right), balancing model complexity and generalization error. (**C**) Predictor variables ranked by importance in the Lasso regression model. Longer bars represent stronger predictors.

The training cohort (*n* = 909) and validation cohort (*n* = 390) exhibited balanced baseline characteristics (Table 1), confirming appropriate data partitioning. In the training cohort, Lasso regression with 10-fold cross-validation (optimal λ = 0.009) identified 17 prognostic variables for model construction (Fig. 2A and B). These included clinical factors (e.g., septic shock, profound neutropenia), infection characteristics (e.g., pulmonary/skin sources, carbapenem resistance), and treatment parameters (e.g., ceftazidime-avibactam [CAZ-AVI]-containing regimens).

**TABLE 1:** Baseline characteristics of the training and validation cohorts^*a*^

Characteristic	Training cohort (*n* = 909)	Validation cohort (*n* = 390)	*P*-value
Age (median [IQR])	41.00 [30.00, 53.00]	46.00 [32.00, 54.00]	0.037
Male (%)	488 (53.69)	210 (53.85)	1
Chronic liver disease (%)	39 (4.29)	21 (5.38)	0.473
Chronic renal disease (%)	8 (0.88)	11 (2.82)	0.016
Diabetes mellitus (%)	100 (11.00)	56 (14.36)	0.107
Primary disease (%)			0.575
AML	487 (53.58)	219 (56.15)	
ALL	273 (30.03)	106 (27.18)	
Others	149 (16.39)	65 (16.67)	
Tumor stage-consolidation (%)	479 (52.70)	206 (52.82)	1
Chemotherapy (%)	770 (84.71)	331 (84.87)	1
HSCT (%)	146 (16.06)	59 (15.13)	0.734
Primary infection (%)			
Primary BSI	512 (56.33)	214 (54.87)	0.672
Pulmonary source	110 (12.10)	47 (12.05)	1
Perianal source	132 (14.52)	65 (16.67)	0.366
Oral source	31 (3.41)	15 (3.85)	0.821
Urinary tract source	4 (0.44)	3 (0.77)	0.742
Skin and soft tissues source	16 (1.76)	5 (1.28)	0.699
Intra-abdominal source	93 (10.23)	41 (10.51)	0.957
Central venous catheter source	12 (1.32)	1 (0.26)	0.144
Concurrent septic shock (%)	56 (6.16)	22 (5.64)	0.815
Concurrent pneumonia (%)	67 (7.37)	26 (6.67)	0.739
Profound neutropenia (%)	653 (71.84)	280 (71.79)	1
Hypoproteinemia (%)	259 (28.49)	99 (25.38)	0.279
Prolonged neutropenia before BSI (%)	77 (8.47)	20 (5.13)	0.047
Prolonged neutropenia after BSI (%)	122 (13.42)	53 (13.59)	1
ESBL (%)	398 (43.78)	157 (40.26)	0.264
Carbapenem resistance (%)	73 (8.03)	31 (7.95)	1
Previously exposed to cephalosporin (%)	272 (29.92)	127 (32.56)	0.379
Previously exposed to PTZ (%)	105 (11.55)	41 (10.51)	0.655
Previously exposed to carbapenem (%)	367 (40.37)	167 (42.82)	0.447
IET48H (%)	63 (6.93)	21 (5.38)	0.36
CAZ-AVI containing regimen (%)	67 (7.37)	21 (5.38)	0.236
LOS (median [IQR])	28.00 [23.00, 40.00]	29.00 [23.00, 36.75]	0.486
Pathogen (%)			0.428
EC	464 (51.05)	189 (48.46)	
KP	445 (48.95)	201 (51.54)	

^*a*^ AML, acute myeloid leukemia; ALL, acute lymphoblastic leukemia; Others, refers to hematological malignancies other than AML and ALL; HSCT, hematologic stem cell transplantation; BSI, bloodstream infections; ESBL, extended-spectrum beta-lactamase; PTZ, piperacillin/tazobactam; IET48H, inadequate empirical therapy within 48 h of the onset of BSI; CAZ-AVI, ceftazidime-avibactam; LOS, lengths of hospital stay; EC, *Escherichia coli*; KP, *Klebsiella pneumoniae*.

Cox regression analysis further identified independent mortality predictors (*P* < 0.1) (Table 2). Mortality in HSCT recipients was 9.59% compared with 5.11% in non-HSCT patients (HR 2.186, *P* = 0.045). Pulmonary source infections had the high mortality (20.38%) compared with non-pulmonary sources (3.59%) (HR 3.661, *P* < 0.001). Infections originating from skin/soft tissue had a mortality rate of 18.75% compared with 5.60% in other sources (HR 3.769, *P* = 0.046). Concurrent septic shock was present in 28.30% of fatal cases, with mortality reaching 26.79% in those with shock, compared with 4.45% without shock (HR 6.216, *P* < 0.001). Concurrent pneumonia increased mortality to 11.94% compared with 5.34% in non-pneumonia cases (HR 3.246, *P* = 0.009). Patients with prolonged neutropenia had a mortality rate of 19.48% (vs 4.57% in those without prolonged neutropenia) (HR 2.988, *P* = 0.002). Carbapenem-resistant infections have a mortality rate of 16.45% compared with 4.90% in non-carbapenem-resistant cases (HR 2.591, *P* = 0.070). Hypoproteinemia showed a trend toward increased mortality (11.97% vs 3.38%, HR 1.802, *P* = 0.056). Notably, the tumor stage-consolidation phase was a protective factor (HR 0.218, *P* < 0.001), as was the use of CAZ-AVI-containing regimens (HR 0.381, *P* = 0.090). CAZ-AVI regimens reduced 30-day mortality in carbapenem-resistant *Enterobacterales* (CRE) BSIs (8.96% vs. 33.33%, *P* < 0.001). To address potential treatment allocation bias of receiving the CAZ-AVI regimen, we performed an IPTW analysis adjusting for key relevant covariates in carbapenem-resistant EC-BSI and KP-BSI patients. After IPTW adjustment, all included covariates were well-balanced (all standardized mean differences < 0.2). The IPTW-adjusted Cox regression demonstrated that the CAZ-AVI-containing regimen was also associated with a 69% reduction in 30-day mortality risk (HR 0.31, 95% CI 0.10-0.96, *P* = 0.041).

**TABLE 2:** Multivariate analysis of all-cause 30-day mortality in HM patients^*a*^

Variable	Z-value	*P*-value	β	HR (95% CI)
Tumor stage-consolidation	−3.817	<0.001	−1.523	0.218 (0.100, 0.477)
HSCT	2.005	0.045	0.782	2.186 (1.018,4.700)
Pulmonary source	5.508	<0.001	1.744	5.718 (3.074, 10.634)
Skin and soft tissues source	1.999	0.046	1.298	3.661 (1.026, 13.062)
Concurrent septic shock	4.787	<0.001	1.827	6.216 (2.942, 13.136)
Concurrent pneumonia	2.623	0.009	1.177	3.246 (1.347, 7.822)
Hypoproteinemia	1.912	0.056	0.589	1.802 (0.985, 3.296)
Prolonged neutropenia before BSI	3.162	0.002	1.095	2.988 (1.516, 5.889)
Carbapenem resistance	1.810	0.070	0.952	2.591 (0.924, 7.262)
CAZ-AVI-containing regimen	−1.697	0.090	−0.965	0.381 (0.125, 1.161)

^*a*^ HM, hematologic malignancies; HSCT, hematologic stem cell transplantation; CAZ-AVI, ceftazidime-avibactam.

### Model construction and validation

**Fig 3:**
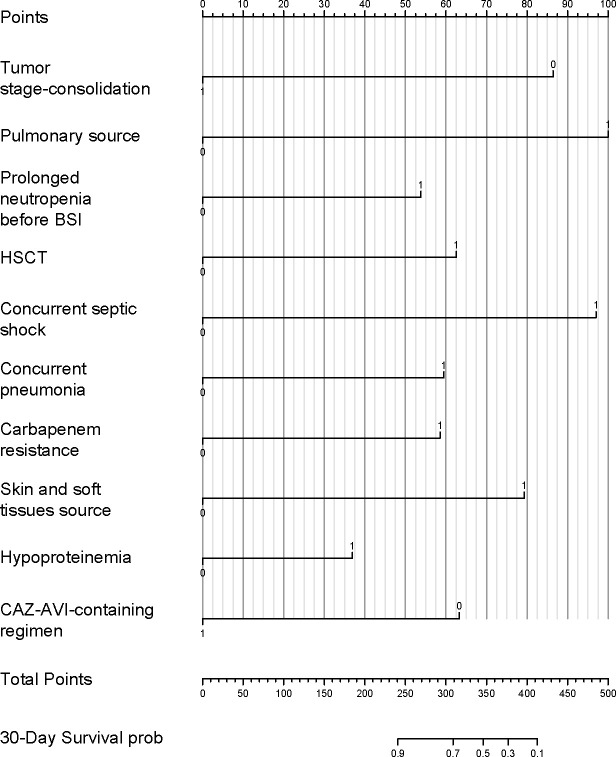
A nomogram estimates the 30-day mortality risk following BSIs in HM patients. For each predictor, locate the value for each patient, draw a line upwards to assign the corresponding points, sum all points on the “total points” axis, and then draw a line downward to determine the estimated 30-day survival probability.

**Fig 4:**
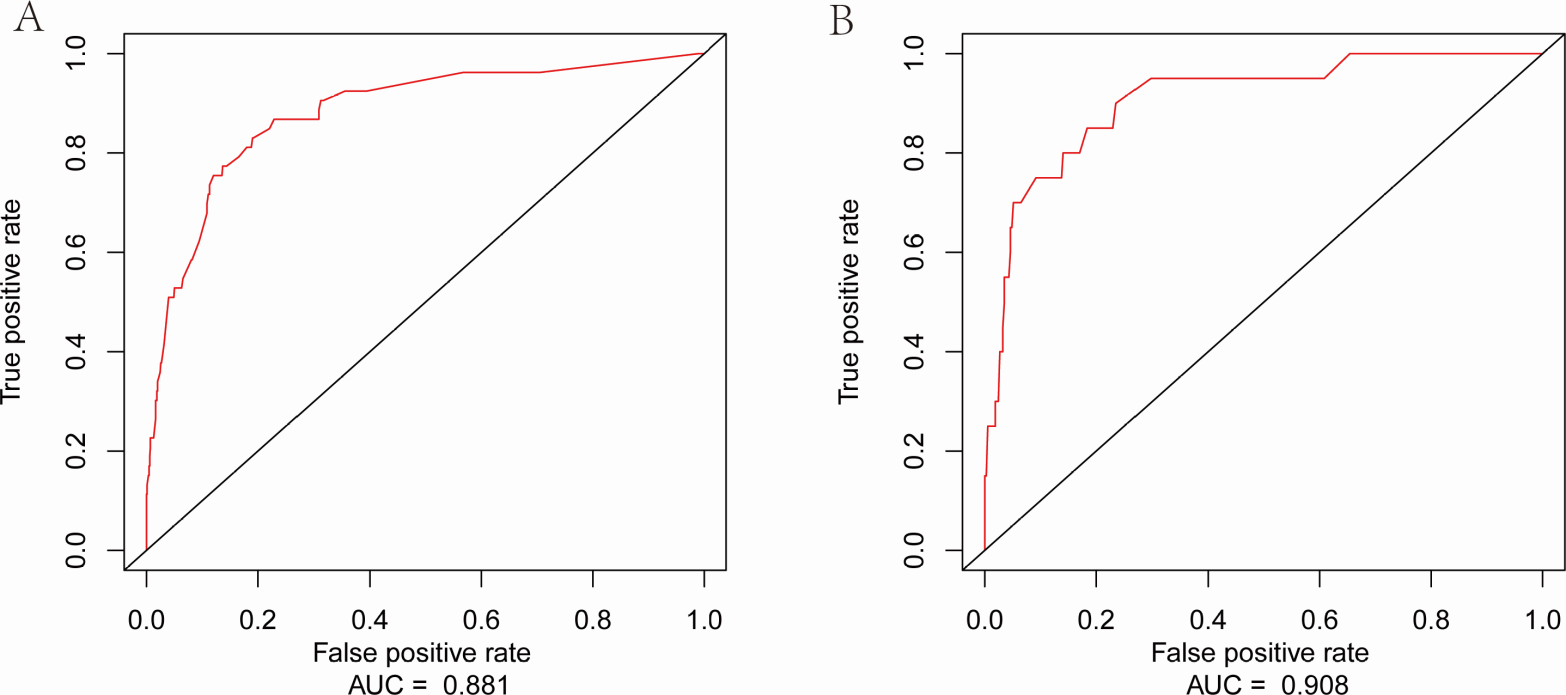
Time-dependent ROC curve analysis of prognostic models. (**A**) Performance in the training cohort (AUC = 0.881). (**B**) Performance in the validation cohort (AUC = 0.908).

A nomogram was created for the purpose of forecasting the 30-day mortality risk in BSI patients, incorporating 10 statistically significant predictive factors (Fig. 3). To enhance clinical applicability, we developed an interactive dynamic nomogram accessible via a web-based interface (https://cuiyuqing.shinyapps.io/DynNomapp/). This tool allows clinicians to input patient-specific variables in real-time and instantly visualize predicted clinical outcomes for bacteremia patients, thereby supporting point-of-care decision-making. The model exhibited excellent performance, with a C-index of 0.872, and internal validation with 100 bootstrap samples yielded a calibrated C-index of 0.834. In the training set, the 30-day mortality prediction had an area under the curve (AUC) of 0.881, while in the validation cohort, it was 0.908 (Fig. 4A and B). The calibration curve showed a high degree of consistency between the 30-day survival probabilities predicted by the model and those actually observed, which was further confirmed by bootstrap validation (Fig. S2A and B).

### Risk stratification and clinical utility

**Fig 5:**
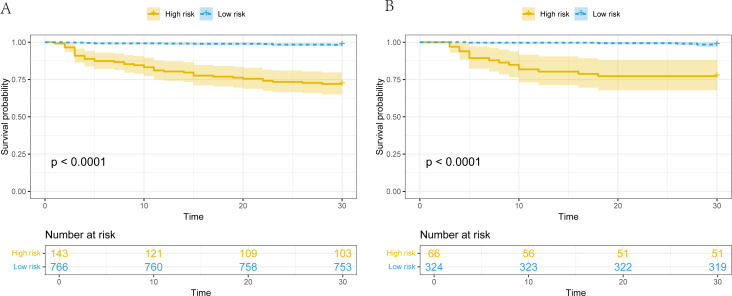
The Kaplan-Meier survival curves showing 30-day mortality for high- and low-risk patients, classified based on the optimal cut-off point. (**A**) Training cohort and (**B**) validation cohort, showing significantly lower survival in high-risk patients (*P* < 0.0001, log-rank test).

Receiver operating characteristic (ROC) curve analysis determined that a cutoff value of 5.08 could effectively differentiate survivors from non-survivors. Kaplan-Meier survival analysis demonstrated robust discriminatory power of the risk stratification model. The high-risk group exhibited a strikingly higher 30-day mortality rate of 28.0% as opposed to the 1.7% in the low-risk group, with a highly significant difference (*P* < 0.001). The prognostic validity was verified in the validation cohort. In this cohort, the mortality rate in the high-risk group was 22.7%, while in the low-risk group, it was 1.5% (Fig. 5A and B). The pronounced divergence in outcomes highlights the model’s ability to identify patients who require intensified clinical monitoring and early intervention. DCA indicated that the model provides superior net benefit compared to strategies that assume all or no patients will die (Fig. S3A and B). Overall, this predictive model proves to be a reliable tool for risk assessment and offers substantial clinical utility.

## DISCUSSION

BSIs are life-threatening complications frequently observed in HM patients, contributing to high mortality rates and a substantial clinical burden. Among the pathogens, gram-negative bacteria (GNB) are the leading cause of BSIs in this population. In our cohort, we identified ten key risk factors associated with 30-day mortality. Notably, treatment with a CAZ-AVI-based regimen and tumor stage-consolidation were independently linked to improved survival. These findings facilitated the development of a prognostic model that can help clinicians in predicting 30-day mortality, thus supporting early intervention and timely decision-making.

The Multinational Association for Supportive Care in Cancer (MASCC) risk index remains pivotal in risk stratification of febrile neutropenia (FN) patients, incorporating variables, including age, disease burden, hypotension, active pulmonary disease, cancer type, dehydration status, and infection site, to categorize patients into high-risk (score <21, requiring hospitalization) and low-risk (score ≥21, eligible for outpatient management) cohorts (13). Only 30% of FN cases achieve microbiologically confirmed diagnoses (14), while BSIs in HM patients exhibit mortality rates of 10% to 34% (2), underscoring the urgent need for refined prognostic models to guide clinical decision-making. Our prior study of 94 CRE-associated BSI cases revealed that 96.8% of patients developed neutropenia (72.3% persisting >14 days), with septic shock (OR = 10.526) and pulmonary infection (OR = 6.289) emerging as independent predictors of 30-day mortality. Notably, CAZ-AVI combined with aztreonam conferred superior survival benefits compared with alternative regimens (OR = 0.068), highlighting the prognostic complexity of antimicrobial resistance and therapeutic selection (15). Wang et al.’s nomogram, which integrates septic shock, refractory malignancy, hypoalbuminemia (<30 g/L), thrombocytopenia (<30 × 10⁹/L), and inappropriate initial antibiotics, demonstrated moderate predictive performance (C-index: 0.870 training, 0.825 validation) ‌ (16). Similarly, a retrospective study determined that the Pitts bacteremia score (with a cutoff of 2.5) was an independent mortality predictor in EC or KP bacteremia cases. Its sensitivity was 83.3%, and specificity was 85.7% (17). However, these models exhibit two major constraints: (i) inadequate sample sizes; (ii) exclusion of carbapenem resistance parameters despite its established prognostic relevance. To address these gaps, our large-scale cohort-derived prognostic model incorporates readily accessible clinical indicators (septic shock, disease status, and hematologic parameters) and integrates carbapenem resistance metrics, thereby enhancing clinical applicability and providing robust evidence for personalized BSI management in hematologic malignancies.

Acute leukemia, including AML and ALL, was the most prevalent HM associated with BSIs in our study. Infection rates during induction chemotherapy for acute leukemia have been reported as high as 86.9% (18). Given the high prevalence of MBL-producing strains in our center (15), the clinical efficacy of CAZ-AVI is mainly attributed to its combination with aztreonam. In our cohort, the 30-day mortality rates for EC-BSI and KP-BSI were 5.62%, which is lower than the 10%–32% that previous studies had reported (2, 5, 19). Mortality among carbapenem-resistant cases was 19.23%, considerably below the 42.5%–74.1% reported for carbapenem-resistant GNB-BSIs (20–22). This supports guideline recommendations prioritizing novel β-lactam/β-lactamase inhibitors for CRE BSIs, especially in immunocompromised hosts requiring rapid pathogen clearance. Furthermore, HM patients in the induction phase or relapsed/refractory stages face a heightened risk of bacterial infections due to factors, such as high tumor burden, exposure to intensive chemotherapy, and underlying immune dysfunction. In contrast, patients in remission exhibit normalized T/B-cell function and restored levels of immunomodulatory cytokines. Prolonged hospitalization and broad-spectrum antibiotic use during relapsed stages may further exacerbate gut microbiota dysbiosis, contributing to adverse infectious outcomes (23).

However, concurrent pneumonia and septic shock emerged as crucial risk factors contributing to increased 30-day mortality in CRE BSI patients, supporting findings from previous research (24). Our study also highlighted BSIs originating from pulmonary sites demonstrated substantially higher mortality compared to other infection foci, a pattern particularly pronounced in patients with HM. In this immunocompromised population, chemotherapy-induced immune suppression and prolonged neutropenia likely exacerbate both infection severity and therapeutic challenges. Furthermore, the development of secondary pneumonia during BSI episodes emerged as an independent prognostic threat, significantly amplifying mortality risk regardless of the primary infection source. Following BSI and the associated systemic inflammatory response, the host initiates a compensatory anti-inflammatory response (CARS), which often leads to widespread immunosuppression. This state is characterized by impaired function of antigen-presenting cells (APCs), such as dendritic cells and monocytes, reduced production of pro-inflammatory cytokines (e.g., TNF-α, IL-12), and persistent release of anti-inflammatory mediators (e.g., IL-10, TGF-β) (25). This immunosuppressive state can also impair innate immunity in the lungs, such as dysfunctional neutrophils being recruited and alveolar macrophages exhibiting weakened inflammasome activation (26). These alterations foster an immunosuppressive pulmonary microenvironment that weakens host defenses, facilitating bacterial colonization and invasion. Moreover, within this environment, certain damage-associated molecules, such as highly sulfated heparan sulfate, may further upregulate virulence factors in opportunistic pathogens (27). The findings highlight the necessity of proactive pulmonary monitoring and preemptive antimicrobial approaches in high-risk cohorts.

Neutrophils play a vital role in the body’s initial defense against bacterial infections, but chemotherapy-induced neutropenia compromises this response. Prolonged neutropenia is a key factor for BSIs among FN patients, with profound impacts on patient outcomes (28). Recent research suggests that neutropenia disrupts the gut microbiome, leading to reduced microbial diversity and prolonged recovery times (29). This highlights the complexity of managing infections in patients with HM, as their immune systems are further impaired by chemotherapy and persistent neutropenia. Additionally, our study identified HSCT, concurrent septic shock, and hypoproteinemia as independent predictors of 30-day mortality. HSCT patients face dual challenges. On one hand, preconditioning regimens required to eradicate bone marrow led to profound and prolonged neutropenia and T-cell dysfunction, critically impairing pathogen clearance capacity (30). On the other hand, prolonged exposure to broad-spectrum antibiotics and chemotherapeutic agents disrupts gut microbiota diversity, promoting colonization by drug-resistant pathogens, such as extended-spectrum beta-lactamase (ESBL)-producing *Enterobacterales*, CRE, and MDR *Pseudomonas aeruginosa* (31). Hypoproteinemia, often a marker of poor nutritional status and liver dysfunction, was strongly correlated with increased mortality. Monitoring and addressing hypoalbuminemia could improve patient outcomes. Septic shock, a severe complication of BSIs, remains a critical driver of mortality (15, 32, 33), and its complex pathophysiology involves a cascade of inflammatory responses leading to multiorgan failure (34). Early recognition and prompt management of septic shock are essential to improving survival in these critically ill patients.

While our study provides valuable insights, external multi-center validation is required to confirm the general applicability of our findings. The local epidemiology, antimicrobial resistance profiles, and clinical practice protocols of the institution may influence the performance of the model. Future research should also center on interventions aimed at mitigating the impact of identified risk factors, particularly among high-risk populations. Nevertheless, our prognostic model offers timely estimates of mortality risk based on easily accessible clinical and laboratory data.

### Conclusion

In conclusion, we identify several key risk factors in our study that are associated with a poor prognosis among patients with *Enterobacterales* BSI, including pulmonary or skin/soft tissue infection sources, HSCT, concurrent septic shock, concurrent pneumonia, hypoproteinemia, prolonged neutropenia before BSI, and carbapenem resistance. Notably, the use of a CAZ-AVI-containing regimen and tumor stage consolidation was the protective factors associated with reduced 30-day mortality. Based on these factors, we developed a prognostic nomogram with strong predictive ability, which serves as a practical tool for clinicians to optimize antimicrobial therapy and improve patient outcomes. However, further external validation through prospective cohort studies remains a key priority for future research.

## Data Availability

The data sets analyzed during the study are deposited in the Mendeley Data repository and can be accessed via DOI: 10.17632/xh2mswykr7.1.

## SUPPLEMENTAL MATERIAL

Supplemental material aac.00973-25-s0001.docx Fig. S1 to S3.

ASM does not own the copyrights to Supplemental Material that may be linked to, or accessed through, an article. The authors have granted ASM a non-exclusive, world-wide license to publish the Supplemental Material files. Please contact the corresponding author directly for reuse.
