# Supplementary material for: Construction and assessment of a predictive framework for Enterobacterales bloodstream infection in real-world adult onco-hematology patients using Lasso-Cox regression
Source: Antimicrob Agents Chemother. 2025 Oct 17;69(12):e00973-25. doi: 10.1128/aac.00973-25 (PMC12691674; doi:10.1128/aac.00973-25)

Figure S1. The Kaplan-Meier survival curves showing 30-day mortality for carbapenem-resistant and non-carbapenem-resistant patients.


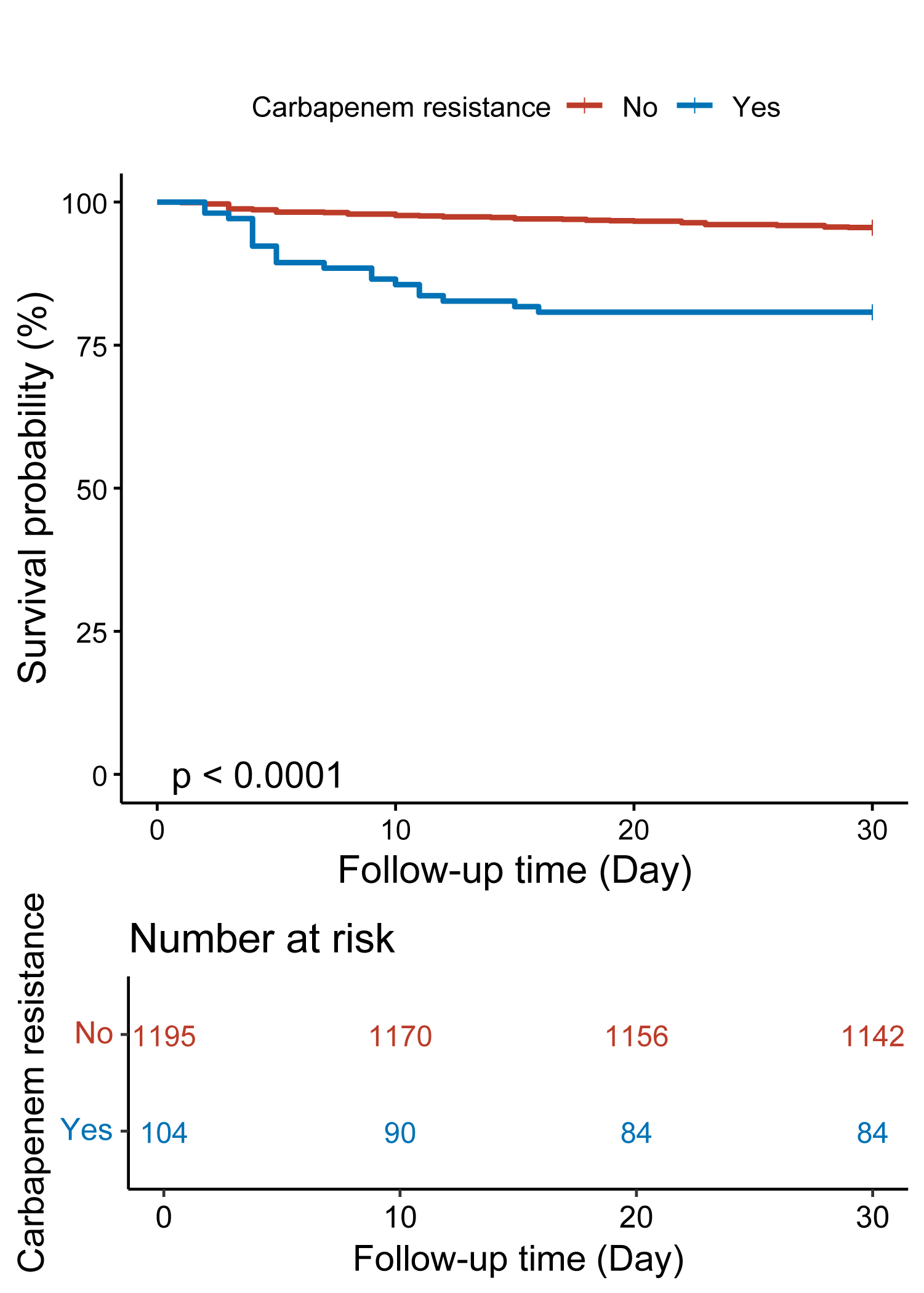


Figure S2. Calibration plot of prognostic nomogram. (A) training cohort. (B) validation cohort. The dashed line represents the ideal perfect prediction, while the solid line depicts the relationship between the model's predicted 30-day survival probability and the actual observed survival rate.


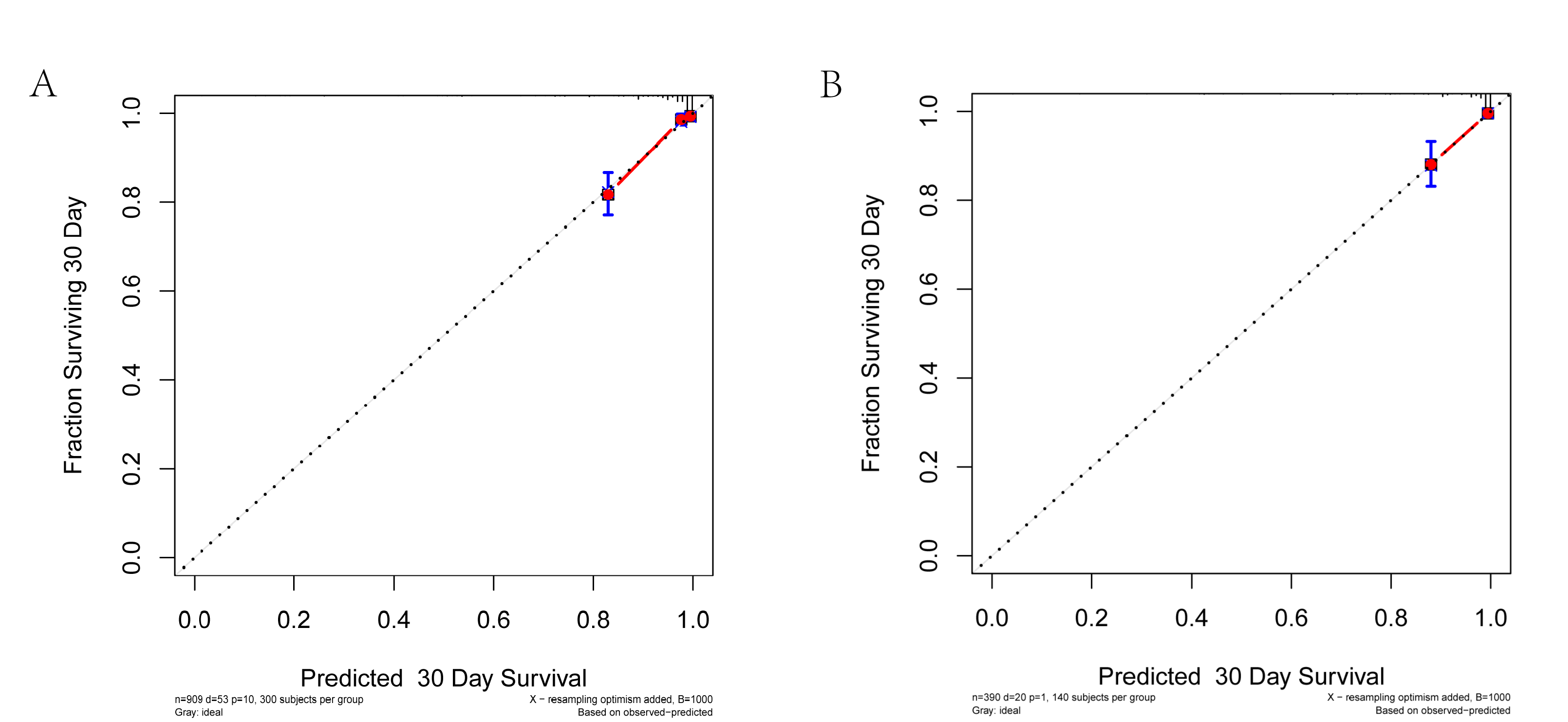


Figure S3. Decision curve analysis (DCA) evaluating the clinical utility of the prognostic model. (A) training cohort. (B) validation cohort. The curve shows that using the nomogram for risk stratification provides a higher net benefit across a wide range of threshold probabilities compared to the strategies of treating all patients or none.


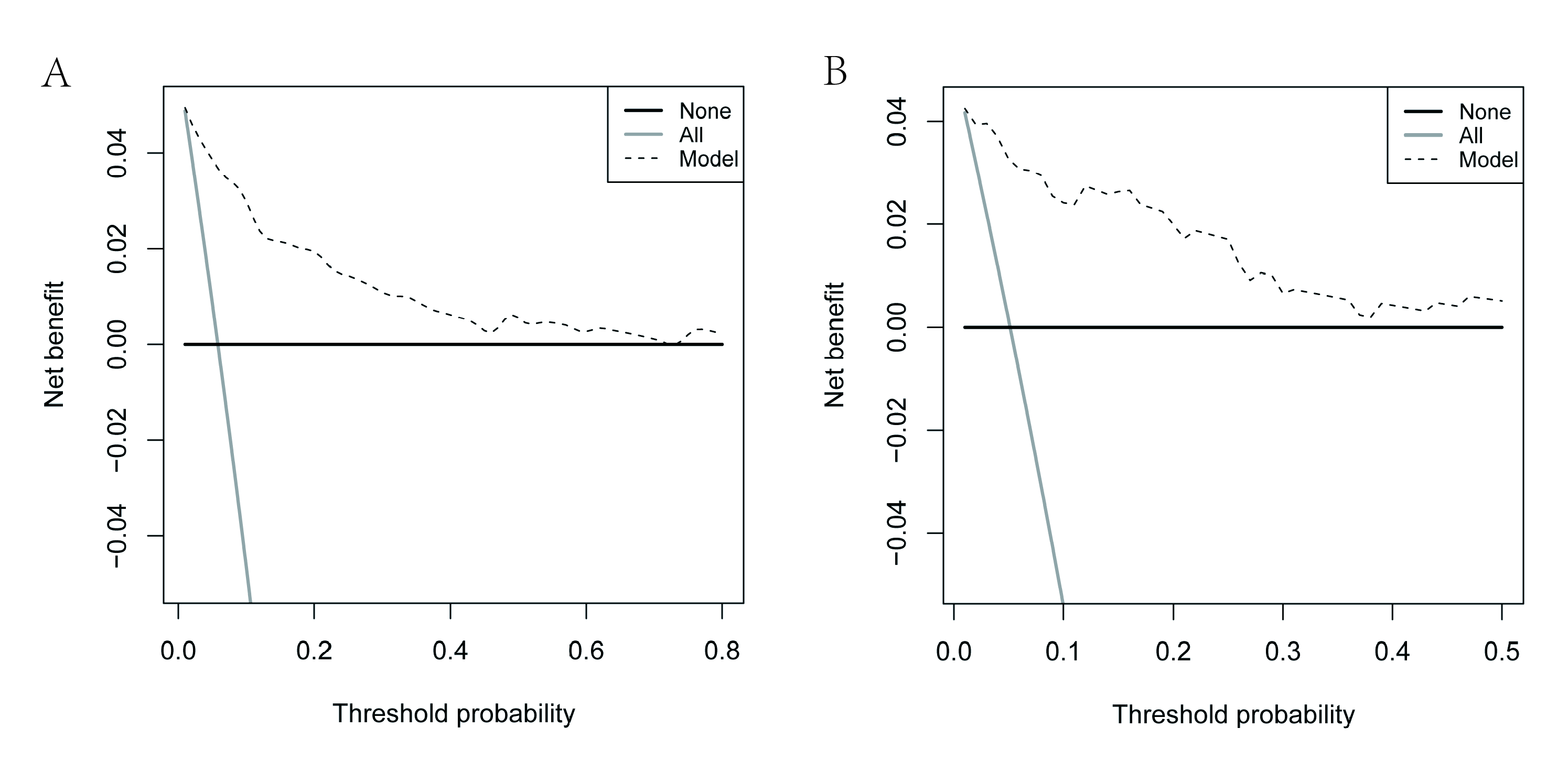

Supplement: Supplemental material — Fig. S1 to S3. [file aac.00973-25-s0001.docx]
